# Using Shear-Wave Elastography to Assess Exercise-Induced Muscle Damage: A Review

**DOI:** 10.3390/s22197574

**Published:** 2022-10-06

**Authors:** Urška Ličen, Žiga Kozinc

**Affiliations:** 1 Faculty of Health Sciences, University of Primorska, Polje 42, 6310 Izola, Slovenia; 2 Andrej Marušič Institute, University of Primorska, Muzejski trg 2, 6000 Koper, Slovenia

**Keywords:** ultrasound, soreness, underlying mechanisms, eccentric exercise, shear modulus

## Abstract

Shear-wave elastography is a method that is increasingly used to assess muscle stiffness in clinical practice and human health research. Recently, shear-wave elastography has been suggested and used to assess exercise-induced muscle damage. This review aimed to summarize the current knowledge of the utility of shear-wave elastography for assessment of muscle damage. In general, the literature supports the shear-wave elastography as a promising method for assessment of muscle damage. Increases in shear modulus are reported immediately and up to several days after eccentric exercise, while studies using shear-wave elastography during and after endurance events are showing mixed results. Moreover, it seems that shear modulus increases are related to the decline in voluntary strength loss. We recommend that shear modulus is measured at multiple muscles within a muscle group and preferably at longer muscle lengths. While further studies are needed to confirm this, the disruption of calcium homeostasis seems to be the primary candidate for the underlying mechanism explaining the increases in shear modulus observed after eccentric exercise. It remains to be investigated how well the changes in shear modulus correlate with directly assessed amount of muscle damage (biopsy).

## 1. Introduction

It is well established that unaccustomed eccentric exercise induces muscle damage [1], which is reflected in loss of maximal voluntary contraction (MVC), evoked force/torque and range of motion (RoM), as well as increased soreness, limb circumference, pressure sensitivity and muscle stiffness [2]. While some degree of muscle damage and the associated inflammation are considered to be paramount for optimal muscle adaptation [3,4], excessive muscle damage impairs athletic performance and makes further training more difficult [5]. An abundant body of research exists on mechanisms underlying muscle damage and its manifestations [6], as well as prevention and treatment strategies [7,8,9].

A plethora of outcome measures have been used to assess the extent of exercise-induced muscle damage [2]. Albeit direct histological examination of muscle samples (biopsy) is considered as the gold standard [10,11,12], it is rarely used in contemporary research. Instead, various proxies for muscle damage are assessed, such as the less-invasive blood marker analysis, or non-invasive assessment maximal strength decrements, RoM loss and pain/soreness [2]. While muscle strength loss is considered by many as the best non-invasive proxy for muscle damage, its manifestation also depends on fatigue and neural factors. Muscle passive stiffness has also been shown to be elevated after damaging exercise [13,14,15,16]. However, older studies used indirect measurements (e.g., passive joint torque) to infer muscle stiffness. Using such measurements is suboptimal, as passive torque is also sensitive to the stiffness of non-muscular tissues (e.g., tendons, ligaments, joint capsule) [17,18]. In addition, passive torque can only provide an estimation of stiffness for the whole muscle group. This is a major limitation in light of the recent evidence of muscle-specific responses to damaging exercise [11,19,20].

**Figure 1:**
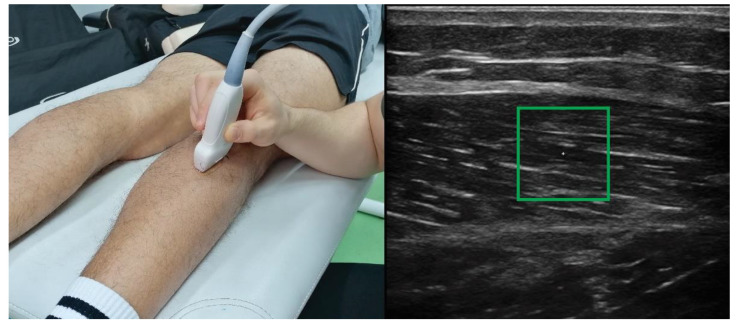
An example of shear-wave elastography use on gastrocnemius muscle. The green box represents the region of interest for which the shear modulus is analyzed.

Recently, ultrasound-based shear-wave elastography has been increasingly used to assess muscle stiffness [21,22,23]. This technique is based on tracking the propagation of acoustic shear-waves, wherein this propagation is faster through stiffer tissues [21]. While qualitative methods are prevalent in some areas of medical diagnostics, the quantification approach for assessment of muscle stiffness is more common. Assuming a constant muscle density (1000 g/dm^3^), shear wave propagation speed is used to calculate the shear modulus (typically expressed in kPa for muscle and tendon tissues) [21,24], which is a measure of the elastic shear stiffness of a material. An example of gastrocnemius lateralis measurement is presented in Figure 1. For exhaustive reviews on shear-wave elastography and its applications to the musculoskeletal system, the readers are referred to other articles [21,22,23,24]. Being non-invasive, reliable [25,26] and operator-independent [27], shear-wave elastography is a promising alternative to assess muscle-specific stiffness associated with exercise-induced muscle damage [18]. While previous reviews provided a broad overview of applications of shear-wave elastography to the musculoskeletal system [21,22,23], there is no literature review to date focusing specifically on the application of shear-wave elastography for assessing muscle damage.

This review aims to summarize the current knowledge of the utility of shear-wave elastography for assessment of muscle damage. When using shear-wave elastography, either shear-wave velocity or shear modulus may be reported. Assuming a constant muscle density, the two measures are directly related [21]; therefore, we will consider them interchangeable in this article. Factors such as exercise and measurement protocol characteristics, muscle group, sex and repeated bout effect will be discussed. We will briefly discuss the effects of different modalities on shear modulus behavior after damaging exercise and conclude with an overview of supposed underlying mechanisms. This review may aid clinicians that want to use shear-wave elastography in assessment of muscle stiffness. Moreover, specific gaps and ambiguities in the literature will be identified, which will provide directions for further research. While other types of elastography have been occasionally used to obtain shear modulus to assess muscle damage [28,29], we choose to focus on shear-wave elastography, as it has been praised for its reliability [23,26,30] and operator independence [27,31,32]. Moreover, virtually all studies published in the last 10 years used shear-wave type elastography to assess muscle damage.

## 2. Changes in Shear Modulus after Damaging Exercise

In general, the literature shows that shear modulus is increased after eccentric exercise. It has been demonstrated that shear modulus is increased immediately or shortly after eccentric exercise for elbow flexors [11,18,33,34,35], elbow extensors [36], hamstrings [19,20], knee extensors [11,37,38,39] and triceps surae muscles [28,40]. Likewise, shear modulus appears to be increased in the days following eccentric exercise in different muscle groups [11,18,20,28,33,38]. However, the results are not entirely consistent, which warrants detailed analysis and discussion. Namely, some studies reported the opposite effect [41], and many studies reported that the increase in shear modulus was dependent on the muscle length during testing [11,18]; also that different synergistic muscles, or even different regions within the same muscle, are not equally affected [11,19,20,38]. Moreover, both increased and decreased shear modulus have been reported after endurance events that presumably elicited muscle damage [42,43,44].

### 2.1. Exercise Characteristics

Studies have predominantly used single-joint isokinetic, or free-weight loads, to induce muscle damage. However, the number of repetitions performed varied from 30 to 150. Lacourpaille et al. [18] used only isokinetic 30 contractions (3 sets of 10 repetitions), performed at 120 °/s, and reported that the shear modulus was increased at 1 h post exercise for biceps brachii and brachioradialis (+42–81%). The shear modulus remained significantly higher even 48 h (+39 %) and 21 days (+14%) after the exercise, for the measurements with the elbow placed in the most extended angle (160°). Another study reported increased shear modulus in the same elbow flexor muscles immediately post exercise and in the following days, using only 36 contractions (3 sets of 12 repetitions), performed with a free weight at 75%1 RM [33]. Triceps brachii shear modulus was also increased (with very large effect size, η^2^ = 0.55) immediately after 40 eccentric contractions with free weights at 80%1 RM [36]. Therefore, it seems that a relatively low number of maximal or submaximal high-intensity eccentric contractions are sufficient to elicit an increase in shear modulus.

Unlike most studies, Kawama et al. [41] reported a decrease in semimembranosus (but not biceps femoris or semitendinosus) shear modulus immediately (3 min) after eccentric deadlifts performed through large RoM. Importantly, no changes in shear modulus were noted after 30 and 60 min, and there were no changes at any timepoint when deadlifts were performed through a narrower RoM (avoiding long muscle lengths), nor following concentric-only deadlifts. It could be that lower intensity used in this study (only 60% of the participant’s body mass) was insufficient to cause notable muscle damage. Indeed, eccentric exercise at a high intensity induces greater muscle damage (evaluated as changes in MVC torque or shear modulus) than exercise at low to moderate intensities [11,45]. Given that no changes in MVC torque were reported by Kawama et al. [41], the absence of muscle damage could explain the decrease in shear modulus in their study. Moreover, as the deadlifts were performed through a large range of motion, stretching-like effect could explain the decrease in shear modulus in semimembranosus. Consistent with this notion, previous studies demonstrated a decrease in hamstring shear modulus after different modes of stretching [46], and the effect seemed to be most pronounced in semimembranosus [47]. Another study failed to find an increase in shear modulus after eccentric exercise was done on trapezius muscle [48]. However, the responses across participants were highly variable and dependent on the location of measurement. In sum, studies that showed increased shear modulus are highly prevalent over studies that showed no changes or the opposite effect. Factors such as exercise intensity [45] and fatigue [49,50] should be taken into consideration when interpreting the discrepancies between studies.

The effect of exercise velocity on shear modulus has rarely been examined, despite the evidence that high-velocity eccentric exercise causes significantly larger reductions in MVC than slow-velocity exercise [51]. Ueda et al. [52] compared the effects of slow and fast velocity eccentric cycling, and while the latter elicited larger decreases in MVC torque, only trends for larger increase in quadriceps shear modulus immediately post-exercise were noted. Further studies are needed to reveal the effects of eccentric exercise velocity on shear modulus of different muscles.

Although most studies used eccentric exercise protocols to induce muscle damage, shear modulus has also been tracked after endurance events that presumably induce muscle damage. Andonian et al. [42] researched the changes in quadriceps shear modulus during and after a 330-km ultra-distance mountain running trail. While there were little changes in shear modulus values from baseline to mid-race (3.56 ± 0.63 and 3.62 ± 0.75 kPa, respectively), a significant decrease was found immediately post-race (3.31 ± 0.61 kPa) with only partial recovery at the 48–72 h measurement (3.45 ± 0.60 kPa). Interestingly, the levels of most muscle damage markers peaked at mid-race (although they were still elevated upon finishing the race). Similar results were obtained by Sadeghi et al. (2018), who measured the shear modulus 24 h and 1 week after short (4 miles), medium (13 miles) or long distance (50 miles) running races. They reported a decrease in shear modulus of the rectus femoris from a mean value of 3.72 m/s before, to a mean value of 2.90 m/s one day after the race for the short distance group. On the contrary, Fouré et al. [43] measured the shear modulus of quadriceps and triceps surae muscles right after short (<60 km) and long (>100 km) running races, and found an increase in the shear modulus in all triceps surae muscles (+5.1–7.0%) after a short race, with possible increases in vastus lateralis and medialis in males only. However, decreased triceps surae shear modulus (−3.1%) was found after longer races. Longer races also elicited larger decreases in MVC, while muscle soreness scores were similar for both distances. It could be that shorter races actually caused greater damage (due to higher exercise intensity), and that extreme fatigue elicited a larger decrease in MVC following longer races. To further complicate the interpretation of the current literature, Sadeghi et al. [44] reported a decrease in shear modulus of the soleus in all three distance groups. They also measured the shear modulus of the biceps femoris and semitendinosus. While the average shear modulus value of the biceps femoris was almost consistent in the short distance group, the value decreased and increased for the middle- and long-distance groups, respectively.

### 2.2. Muscle Group

Several studies have shown that increases in shear modulus after eccentric exercise are not necessarily uniform across synergist muscles. For instance, Green et al. [28] reported that soleus shear modulus was increased immediately after 15-min backward walking exercise, but not after 48 h. Conversely, gastrocnemius shear modulus only tended to be increased immediately after the exercise, and it was then statistically significantly increased at 48 h after exercise. Leung et al. [40] reported a large increase in shear modulus in gastrocnemius (+75 ± 47.7% in medial head, +71.7 ± 51.8%) after 150 eccentric heel drop contractions, but did not assess the soleus muscles. The discrepancies between the studies could be explained by the type of exercise, as heel drops likely placed a significantly higher load on the gastrocnemius. At the same time, different levels of fatigue could cofound the results, as fatigue was shown to decrease shear modulus [49,50].

In addition, several studies reported that biarticular muscles (rectus femoris and biceps brachii) were more affected compared to monoarticular muscles (brachialis, vastus lateralis, vastus medialis) [11,37,38,39,53]. For instance, Lacourpaille et al. [11] reported approximately twofold higher shear modulus in rectus femoris and biceps brachii, compared to their monoarticular synergists. These inter-muscular discrepancies are in accordance with larger edema observed in biarticular muscles after eccentric exercise [54,55]. It could also be that biarticular muscles are damaged more due to the higher percentage of fast-twitch muscle fibers, which are believed to be more prone to damage than slow-twitch fibers [54,56,57]. Xu et al. [38] have also shown a notable increase in rectus femoris shear modulus, immediately (+37.3%) and 48 h (+22.4%) after eccentric exercise, with only slight changes (<10%) in the vastus lateralis and vastus medialis muscles. They suggested the smallest cross-sectional area of recuts femoris among the three muscles [58] could make it more prone to damage. Given that muscle damage is dependent on fascicle strain [59], the vastii could also be at lower risk for damage because of their higher pennation angle compared to rectus femoris [60].

For hamstrings, two studies reported that the shear modulus for semitendinosus and biceps femoris were increased after eccentric exercise, while the shear modulus of semimembranosus was not [19,20]. In both studies, semitendinosus was also more affected than biceps femoris. In addition, Goreau et al. [19] reported that the amount of damage indicated by shear modulus change was correlated with muscle activation during exercise. Notably, both studies used knee-dominant exercises (i.e., both studies used single-joint isokinetic contraction, while Voglar et al. [20] also used Nordic hamstring exercise). Although no differences between muscles were found regarding average muscle activation during the damaging exercise in the Goreau et al. study [19], knee-dominant exercises are known to preferentially target semitendinosus, while hip-dominant exercise preferentially stresses semimembranosus and biceps femoris [61]. Future studies are needed to reveal if shear modulus would increase more in semimembranosus after hip-dominant eccentric exercises. If this is the case, the differences in responses of individual hamstring muscles would be at least partially explained by exercise selection.

### 2.3. Muscle Length

Muscle length achieved during eccentric exercise seems to be positively associated with the amount of muscle damage [62,63]. Accordingly, a recent study reported greater changes in rectus femoris shear modulus after eccentric exercise in a supine position (larger muscle length) compared to sitting position (shorter muscle length), with similar differences between positions also observed for reductions in MVC torque [64]. It is conceivable that in the sitting position (rectus femoris being shortened over the hip), the fascicle strain was lower (i.e., the muscle-tendon elongation being achieved predominantly through tendon elongation) compared to a supine position. Indeed, fascicle strain is related to the amount of muscle damage [59]. Moreover, when muscles are already significantly stretched at the beginning of the contraction, a greater portion of the movement will be performed at the descending limb of the force-length relationship. This could explain the results of the aforementioned study [64], as sarcomere disruptions have been shown to be more prevalent on the descending than ascending limb of the force-length relationship [1]. Future studies are needed to verify if changes in shear modulus are dependent on muscle length achieved during eccentric exercise.

Several studies have indicated that an increase in shear modulus after eccentric exercise is highly dependent on muscle length during testing, with larger changes observed when muscles are in longer positions [11,18,35,38,65]. This could indicate that increase in shear modulus reflects the disturbances of Ca^2+^ homeostasis, given that muscles’ sensitivity to Ca^2+^ is increased with muscle elongation [66,67] (see the “Underlying Mechanisms” section for further discussion on mechanisms involved in increased shear modulus after damaging exercise). In addition to showing that changes in elbow flexors and knee extensors shear modulus were higher in lengthened muscle after eccentric exercise, Lacourpaille et al. [18] also reported a decreased slack length in biceps brachii (inferred from changes in shear modulus as the muscle was stretched) and higher shear modulus through the whole RoM beyond slack length. The authors attributed this increase to titin (a protein connecting Z line to the M line in the sarcomere), which modulates passive muscle force in a Ca^2+^-dependent manner [68]. In sum, it seems that muscles must be put at least somewhat beyond their slack length in order to observe changes in shear modulus after eccentric exercise. Namely, when muscles are shortened, increases in shear modulus are often not evident at all [11,38].

### 2.4. Sex Differences

While animal studies suggest higher susceptibility to muscle damage in males compared to females [69], the results are equivocal in humans [70,71,72,73,74]. Only a handful of studies have compared changes in shear modulus between sexes. After free-weight eccentric elbow flexion contraction, both males and females showed the increase in shear modulus, however, the peak increase was more pronounced in males (+33.3%) than females (+14.3%), which seems to be related to a higher inflammatory response in males [33]. After short and long endurance trail competitions, the triceps surae shear modulus increased and decreased, respectively, with no sex differences [43]. Baseline sex differences should be considered in future studies, as higher pre-exercise shear modulus before the exercise is correlated with higher muscle damage (i.e., higher voluntary torque reduction) after the exercise [38]. Indeed, males had slightly higher baseline shear modulus in the study by Agten et al. [33], however, the sample sizes are too small (5 males, 5 females) to draw any meaningful conclusions. Future studies examining sex differences should consider including baseline shear modulus as covariate in their analyses. In addition, training status should also be considered, as it may affect the sex differences regarding the responses to eccentric exercise [74].

### 2.5. Repeated Bout Effect

Recently, studies have begun to investigate if shear modulus could also provide insights into the repeated bout effect (a phenomenon characterized by less muscle damage incurred during a second, subsequent bout of eccentric exercise [75]). Previously, it has been reported that passive muscle stiffness inferred from passive joint torque on dynamometer was sensitive to the repeated bout effect [15]. Chalchat et al. [37] reported a lower amount of muscle damage (assessed by soreness scores, pressure sensitivity, muscle thickness, MVC and twitch torque, doublet amplitudes at 10 and 100 Hz, as well as RoM) to recuts femoris and vastus lateralis after the second bout of downhill walking, and similar was found for shear modulus. In fact, the shear modulus did not statistically significantly increase after the second bout (relative to baseline values before the second bout). Interestingly, recuts femoris shear modulus remained increased until the second bout (14 days in-between bouts), and the amount of this increase was correlated with the index of protection regarding the rate of torque development and doublet amplitude at 10 Hz. Moreover, the higher the increase in shear modulus was in the first bout, the lower the increase in the second bout (for both rectus femoris and vastus lateralis). This could indicate that muscles becoming stiffer is not only a reflection of damage, but also a possible protective mechanism, perhaps related to extracellular matrix remodeling [76]. A study by Ema et al. [64] assessed the repeated bout effect on the quadriceps muscles, and also reported that rectus femoris (both in proximal and distal regions) shear modulus was less affected after the second bout of eccentric exercise. The magnitude of protective effect was the same regardless of the muscle length in the first bout (the participants performed the exercise in the first session in either sitting or supine positions). Thus, this study did not support the hypothesis that larger changes in shear modulus in the first bout translate into larger protective effect. Future studies, preferably including the assessment of histological changes, are needed to reveal the influence of shear modulus changes on subsequent repeated bout effect.

## 3. Correlations between Changes in Shear Modulus and Other Proxies of Muscle Damage

To the best of our knowledge, no study to date has assessed the correlation between changes in shear modulus and changes in biopsy-based measures of muscle damage. Thus, it remains unknown to what extent the increase in shear modulus actually reflects the degree of muscle damage. However, some preliminary insights can be gained from studies that correlated shear modulus with other indirect measures of muscle damage. For instance, a recent study has reported that relative EMG activation of semitendinosus after eccentric exercise was related to the extent of damage indicated by shear modulus (r = 0.69) [19].

Most authors agree that MVC force/torque loss is the best indirect measure of muscle damage [77,78]. After trail running endurance events, there were no correlations between the changes in shear modulus (triceps surae and quadriceps muscles) and MVC torque [43]. In addition, no significant correlations were reported between the recovery rate of MVC and shear modulus for the knee extensors after eccentric exercise [53]. On the contrary, Heales et al. [39] reported a significant correlation between increases in rectus femoris shear modulus and the loss of MVC torque (r = −0.65). In addition, Lacourpaille et al. [11] reported a strong correlation between the increase in shear modulus of the knee extensors and elbow flexors at 30 min after the eccentrics exercise and MVC torque loss at 48 h in the respective muscles (r = −0.82 and −0.80). Similar results for the elbow flexors only were reported by Jones et al. [79] (r = −0.72). In sum, it seems that changes in shear modulus could be related to MVC force/torque losses. The study by Lacourpaille et al. [11] further suggests that early increase in muscle shear modulus can predict MVC loss later on, which has significant implications for practical work in sport and physical therapy.

Studies have also looked into a possible relationship between changes in shear modulus and changes in various blood markers that presumably reflect the degree of muscle damage. Andonian et al. [42] reported negative correlations between the percent change in shear modulus and the corresponding percent changes in creatinine, glomerular filtration rate, plasma osmolality, and white blood cells and neutrophils concentration after 330-km ultramarathon running race. However, all correlation coefficients were very small (r ≤ 0.28). Another study reported somewhat higher negative correlations (ρ = −0.51 to −0.56) between the decreases in triceps surae and quadriceps shear modulus and increases in blood markers and creatin kinase activity after trail running events [43]. On the contrary, Chalchat et al. [37] did not find a correlation between changes in quadriceps shear modulus and changes in creating kinase values after eccentric exercise (downhill walking). Moreover, Hicks et al. [80] reported no correlation between changes in patellar stiffness and the change in CK values after eccentric exercise.

Muscle thickness has been suggested as another surrogate measure for muscle damage, expecting to be increased as a consequence of edema [81]. However, studies researching the relationship between changes in shear modulus and changes in muscle thickness (triceps brachii or the upper trapezius) did not report any statistically significant correlations [36,48]. Furthermore, soreness of the quadriceps muscles was not in correlation with the shear modulus changes after eccentric exercise [37,38]. Only Andonian et al. [42] reported a small negative correlation between leg pain and the shear modulus during the 330 km race (r = −0.28).

Three studies concomitantly assessed the changes in joint RoM and shear modulus, but only Kawama et al. [41] reported no correlation between the knee joint RoM and the change in shear modulus of the long head of biceps femoris, semitendinosus or semimembranosus immediately after deadlift exercise. The other two studies did not give any information about the correlation between RoM and shear modulus. In one study, the changes in shear modulus preceded the changes in RoM [37], while the other reported very similar patterns for RoM and shear modulus changes over multiple assessment timepoint, and also similar effects of HMB supplementation on both measures [35]. In principle, increased shear modulus should be associated with lower RoM, however, future studies are needed to confirm this. Interestingly, Voglar et al. [20] reported an increase in hamstring shear modulus (immediately, 1 h, and 24 h after eccentric exercise), but no changes in passive knee torque. However, as mentioned before, passive joint torque measurements also reflect the stiffness of tendons and other soft tissue surrounding the joint [17,18], which could explain these results.

Overall, only MVC loss and certain blood biomarkers are possibly related with shear modulus changes after damaging exercise, however, even for these two the findings are mixed and likely depend on exercise type. There is little evidence to support the association between muscle soreness, muscle thickness and joint RoM with changes in shear modulus. Additional studies are urgently needed to investigate how well the shear modulus reflects the actual extent of muscle damage.

## 4. Effects of Different Modalities on Shear Modulus after Eccentric Exercise

A handful of studies have investigated the effects of different modalities aimed to ameliorate the increase in shear modulus after eccentric exercise, including vibration [34,79], compression garments [82] and β-Hydroxy-β-Methylbutyrate (HMB) ingestion [35,65]. Pournot et al. [34] reported no effect of vibration applied to hand-held vibrator to biceps brachii muscle; regardless of the vibration application, the shear modulus was increased immediately and 5 min after the exercise. Likewise, Jones et al. [79] reported no effect of whole-body vibration application on biceps brachii shear modulus at 24 h, 48 h and 1 week after eccentric exercise, however, the vibration group reported less soreness and had higher MVC after 24 h. This is in accordance with previous studies showing that vibration may ameliorate strength loss after eccentric exercise [83,84], while other measures such as thigh circumference [83] are not affected. It has been suggested that vibration primarily affects proprioceptive and neuro-muscular function, which reflects in increased strength, but does not affect the extent of muscle damage [85].

Heiss et al. [82] reported that wearing compression garments completely attenuated the changes in gastrocnemius shear modulus after eccentric exercise (150 heel drops), while a decrease was found in the non-compressed condition. These results are difficult to interpret for two reasons: (a) because all the remaining outcome measures (range of motion, soreness, calf circumference, intra-muscular edema) were unaffected by compression; and (b) because the decrease in shear modulus is not consistent with other studies. Large RoM during heel drop could induce effects similar to stretching (which would be consistent with Kawama et. al. [41] who used deadlifts and reported a decrease in shear modulus). However, it remains ambiguous why compression prevented the decrease in shear modulus. Since the literature generally supports the benefits of compression garments for recovery [86,87], further studies are clearly needed to elucidate the effects of compression garments on shear modulus behavior after eccentric exercise.

Two studies [35,65] investigated HMB supplementation and reported that both 2-week and 4-week supplementation prior to elbow flexor eccentric exercise attenuated MVC and RoM loss, while the only effect on shear modulus was seen immediately post-exercise. While authors agree with the notion that Ca^2+^ release during or following damage to the muscle membrane due to breakdown of the sarcomere causes an increase in shear modulus [6,11], further investigation would be needed to reveal why shear modulus was affected by HMB only immediately after the exercise, and to explain the discrepancy between shear modulus and RoM outcomes. The same research group also investigated fish oil supplementation and reported that it ameliorated the loss of MVC, RoM and also the increase in biceps brachii shear modulus [88].

## 5. Underlying Mechanisms

The currently prevailing hypothesis is that increases in shear modulus after damaging exercise are closely related to disturbance of Ca^2+^ homeostasis [11,89]. During subsequent eccentric contractions, the damage is ultimately spread enough to cause damage to the muscle fiber membrane [90] and disruption to structural muscle proteins [10,77]. The subsequent increase in intramuscular Ca^2+^ concentration is thought to trigger an increase in the number of stable attached cross-bridges (also termed as “contracture clots”) [11,18,89]. In addition, increased Ca^2+^ could augment the binding between titin and actin proteins, further increasing muscle stiffness [11,68,91]. Larger increases in shear modulus following damaging exercise at long (compared to short) muscle lengths [18,38] also support this hypothesis, as muscle sensitivity to Ca^2+^ is increased at longer lengths [66]. However, it should be noted that no study to date has assessed the relationship between shear modulus and sarcoplasmic Ca^2+^; thus, this hypothesis cannot be conclusively confirmed.

Some authors have proposed that increased shear modulus could also be a reflection of inflammation and edema [1,13,28,33]. However, increases in shear modulus seem to precede the increases in edema [18], meaning that edema could potentially explain only a portion of the increase in shear modulus in later stages. Such a notion was also proposed by Chalchat et al. [37], who observed increased shear modulus and muscle thickness for more than 48 h after eccentric exercise, while excitation–contraction coupling (a mechanism highly reliant on Ca^2+^) was already restored to baseline. Therefore, it could be that early increases in shear modulus are primarily reflecting the disruption of Ca^2+^ homeostasis, while increased edema and inflammation are also important determinants later on. However, this remains purely speculative. It should also be noted that increases in shear modulus do not appear to be correlated to increases in muscle thickness [36], which is one of the proxy measures for edema. Moreover, Hotfiel et al. [92] observed a decreased shear modulus and increased edema after their eccentric exercise protocol, further questioning the relation between the two outcomes.

Decreases in shear modulus observed in some studies suggest that various opposing mechanisms could underline muscle stiffness changes after damaging. Indeed, muscle fatigue results in decreases in shear modulus [49]. This could explain why endurance events, while damaging to the muscles, caused an overall decrease in shear modulus. In addition, it was suggested that a loss of cytoskeletal desmin (a protein connecting adjacent myofibrils to the Z-lines) could contribute to decreases in muscle stiffness [11,93]. Further studies utilizing histological examinations are needed to reveal the intricate interplay between various determinants of muscle stiffness after eccentric exercise.

## 6. Limitations and Future Directions

In general, this review suggests that shear modulus, obtained by shear-wave elastography, is sensitive to exercise-induced muscle damage. However, some limitations need to be stressed. Shear modulus is very sensitive to probe orientation, location and applied pressure [26,94,95,96]. While these variables were carefully standardized throughout most of the included studies, this might be more difficult to achieve in everyday practice, which would allow for (albeit likely small) errors. In addition, smaller changes in shear modulus (e.g., when muscle damage is mild) might be difficult to detect. The literature lacks investigations of direct relationships between direct measurements of muscle damage and shear modulus. Thus, it is not clear if shear modulus can replace direct assessments altogether. Future studies should concomitantly assess the changes in direct markers of muscle damage and shear modulus. Furthermore, the sensitivity of the shear modulus to exercise bouts of varying volume, type and intensity should be further evaluated.

## 7. Conclusions

In general, the literature supports the shear-wave elastography as a promising method for assessment of muscle damage. Most studies report increases in shear modulus immediately and up to several days after eccentric exercise. Moreover, it seems that shear modulus increases are related to MVC loss. We recommend that shear modulus is measured at multiple muscles within a muscle group and preferably at longer muscle lengths. While further studies are needed to confirm this, the disruption of Ca^2+^ homeostasis appears as the primary candidate for the underlying mechanism explaining the increases in shear modulus observed after eccentric exercise. It remains to be investigated how well the changes in shear modulus correlate with muscle damage assessed by direct gold standard methods (muscle biopsy).

## Data Availability

Not applicable.
